# Genetic structure and temporal environmental niche dynamics of sideoats grama [*Bouteloua curtipendula* (Michx.) Torr.] populations in Mexico

**DOI:** 10.1371/journal.pone.0254566

**Published:** 2021-07-15

**Authors:** Alan Álvarez-Holguín, Carlos Raúl Morales-Nieto, Raúl Corrales-Lerma, Jesús Alejandro Prieto-Amparán, Federico Villarreal-Guerrero, Ricardo Alonso Sánchez-Gutiérrez

**Affiliations:** 1 Campo Experimental La Campana, Aldama, Instituto Nacional de Investigaciones Forestales, Agrícolas y Pecuarias (INIFAP), Chihuahua, Mexico; 2 Facultad de Zootecnia y Ecología, Universidad Autónoma de Chihuahua, Chihuahua, Chihuahua, Mexico; 3 Facultad de Ciencias Agrotecnológicas, Universidad Autónoma de Chihuahua, Chihuahua, Chihuahua, Mexico; 4 Campo Experimental Zacatecas, Calera de Víctor Rosales, Instituto Nacional de Investigaciones Forestales, Agrícolas y Pecuarias (INIFAP), Zacatecas, Mexico; Brigham Young University, UNITED STATES

## Abstract

In the past years, several plant breeding programs have been done to select outstanding genotypes of sideoats grama (*Bouteloua curtipendula*) for restoration purposes. Such programs have been focused mainly on agronomic traits; however, little attention has been paid to the genetic structure and environmental adaptation of the selected genotypes. Thus, in this study we evaluated the genetic structure of 85 sideoats grama populations in Mexico. In addition, we modeled the past, present and future environmental niche of the genetic clusters of this species. Ninety sideoats grama populations were genetically analyzed through AFLP (Amplified Fragment Length Polymorphisms) markers. The environmental niche of the population clusters was modeled by using the maximum entropy method. The genetic analysis separated the populations into two genetically different clusters (p = 0.0003). The differentiation of these lineages can be partially explained by the paleoclimatic events experienced during the last interglacial and glacial maximums. Consequently, the genetic clusters have different environmental niche at the present time. Suitability areas for the distribution of Cluster I are mainly located in the central part of the country while the environmental niche of Cluster II is located in the semiarid region, close to the mountain range of the Sierra Madre Occidental. Thus, selection and restoration programs with sideoats grama must be carried out using local germplasm from each environmental niche. Given the environmental niche of both genetic clusters will suffer changes in the near and mid-century future, climate change must be considered for genotypes selection and restoration programs.

## Introduction

Sideoats grama [*Bouteloua curtipendula* (Michx.) Torr.] is one of the most utilized species for grasslands restoration in northern Mexico. This grass has been widely used due to its great adaptability to a wide range of environmental conditions and due to its high forage quality [1]. In the past years, breeding programs have focused on selecting outstanding genotypes for restoration of degraded grasslands [2–5]. However, these selection programs have mainly focused on agronomic traits, such as high biomass and seed production, and the pressure of these selections may lead to genetic depression and loss of traits related to environmental adaptation [6, 7]. Thus, it is fundamental to know about the genetic structure and environmental adaptation of the selected genotypes.

Genetic structure is an important factor influencing the adaptation to environmental conditions and the distribution of grass species [7]. For this reason, knowledge about the genetic structure is crucial to design strategies for the conservation and utilization of native germplasm. To assess the genetic structure patterns and quantify the number of genotypes existing within the species, a diverse array of neutral molecular markers, i.e. RAPDs, AFLPs or microsatellites have been used [8]. Data of genetic structure may be useful to model the migration patterns shown by different species through time and, to determine the interactions that have influenced the genetic structure within the species [9]. In addition, knowledge about the genetic structure allows identification of present and future suitable areas for the use of a given genotype in ecological restoration [10]. In turn, this may help to increase the probability of success of restoration programs [11, 12].

A way to determine suitable areas for the use of a genotype is through the use of mathematical models [13]. The maximum entropy method is a mathematical model widely used to estimate the environmental niche of species of flora and fauna [14, 15]. The maximum entropy method is available in the MaxEnt software and has several advantages, i.e., it is possible to obtain a high level of certainty using limited records of presence [16]. Hence, it serves to determine the environmental niche of different genotypes, if their geographic distribution is known [17, 18]. Furthermore, this model has been used to identify suitable areas for distribution and use of sideoats grama in Mexico and the United States [19, 20]. Thus, the objective was to evaluate the genetic structure of sideoats grama populations in Mexico and to model the past, present and future environmental niche of the genetic clusters of this species.

## Materials and methods

### Sampling

**Fig 1:**
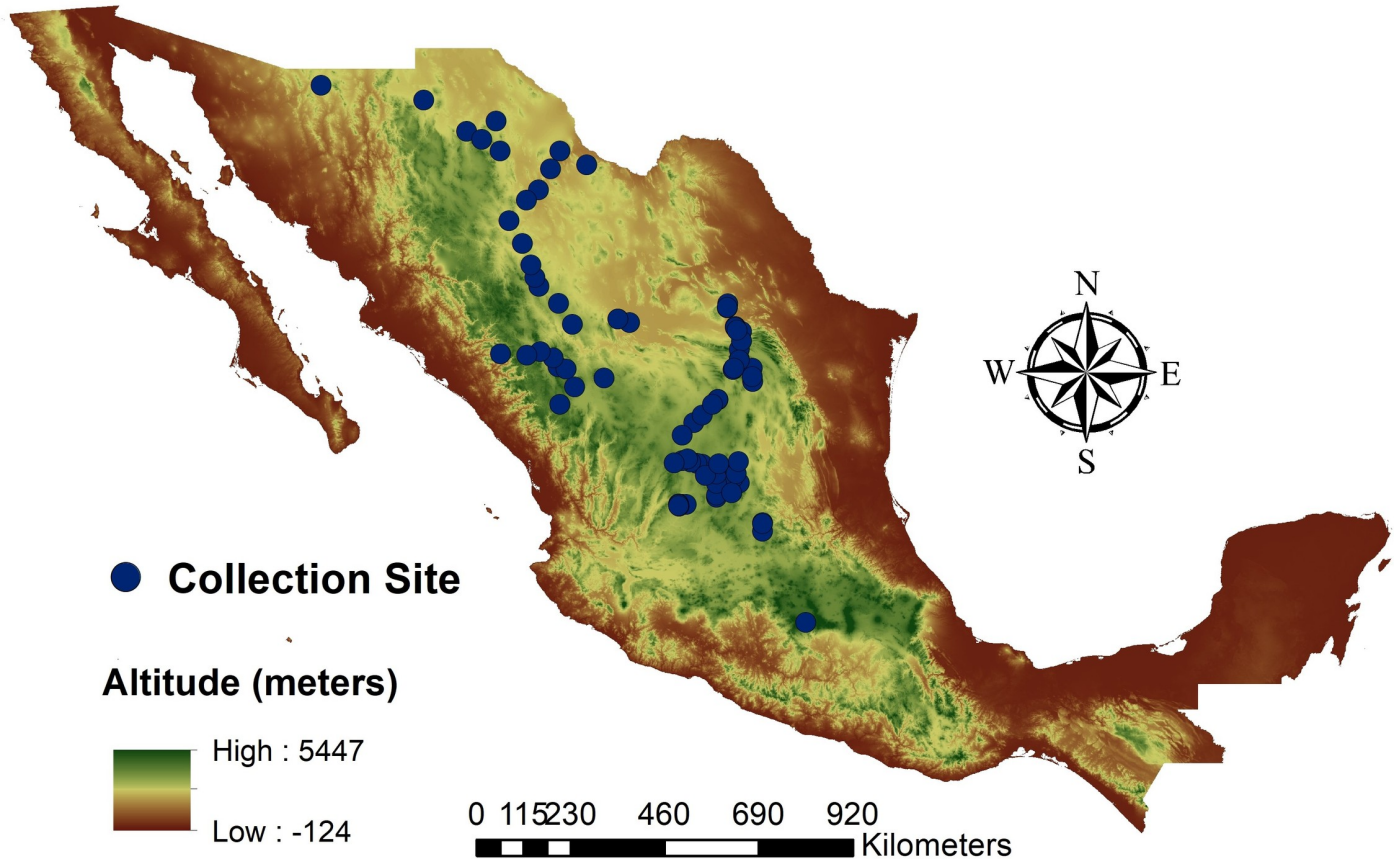
Sites geographic location of the collected samples from the 85 sideoats grama (*Bouteloua curtipendula*) populations. Digital Elevation Model from INEGI (https://www.inegi.org.mx).

In this study, 85 natural populations, distributed in 10 states of Mexico, were sampled (S1 Table). Such populations are representative of the species environmental and habitat diversity in Mexico. This species has a distribution of 548,719 km2 [20] and the sampling area represents 57% (316,740 km2) of the national distribution. Leaves from at least three plants per population were collected and stored in coolers with dry ice. The samples were then transported to a laboratory for further genetic analysis. Sampling was performed in the arid and semiarid regions of the country, based on the sideoats grama distribution shown in Fig 1.

### Genetic structure analysis

The genetic structure of the sampled sideoats grama populations was analyzed trough AFLP (Amplified Fragment Length Polymorphisms). Leaf samples were used for the DNA extraction, which was performed following the method proposed by Doyle and Doyle [21]. DNA from the three sampled plants from each population was bulked for the analysis. AFLP protocol was carried out following Vos et al. [22]. It started with restriction digestion of 2 ml of diluted DNA, using the EcoRI and MseI restriction enzymes. The digested DNA fragments were then ligated to EcoRI and MseI adapters. The pre-amplification step was carried out with AFLP primers having an additional single nucleotide (EcoRI + AandMseI + A). For the selective amplification, the fluorescent-labeled primer combinations EcoRI + AGG–MseI + CTG; EcoRI + AGG–MseI + CTA; EcoRI + AGG–MseI + CGG; EcoRI + ACA–MseI + CGA and EcoRI + ACA–MseI + CAC were used. Polymerase chain reaction (PCR) was performed in a thermal cycler ERICOMP TwinBlock using the following amplification profile: 1 cycle with a duration of 30 s at 94°C, 30 s at 65°C and 1 min at 72°C, 12 cycles of 94°C for 30 s, 65°C for 30 s (with decrements of 0.78°C at each cycle), 72°C for 1 min and 23 cycles of 94°C for 30 s, 56.8°C and 72°C for 1 min. The selective amplification products (5 μL) were mixed with 2 μL of 5X SGB (buffer: 95% formamide, 0.05% bromophenol blue, 0.05% cyanol xylene and 10 mM NaOH). The DNA was denatured at 95°C for 10 min. Finally, the AFLP fragments were analyzed by electrophoresis, which was performed in an acrylamide gel at 6.5%. The DNA bands were transferred from the gel to a nylon membrane (0.45 μm of 30×39 cm), for 16 h with the Southern Dry Blot technique. The membranes were exposed 3 to 4 h to a radiographic film. Then AFLP data from all primers was transferred into a binary matrix, scored as “1” (presence of fragment) and “0” (absence of fragment). Bands were considered polymorphic if the frequency of one of its states (present or absent) was less or equal to 0.98 (present or absent in at least 84 from the 85 populations.

The binary data were subjected to a genetic structure analysis, based on Bayesian clustering algorithms. This analysis was performed in the STRUCTURE version 2.3.4 software [23, 24]. Correlated allele frequencies and admixed model were applied with 100,000 burn-in and 10,000 Markov chain Monte Carlo (MCMC) replications. The predefined number of clusters (*K*) was from 1 to 10. Thirty independent simulations were conducted for each *K* value. Structure Harvester was used to estimate the optimum value for *K* [25] (http://taylor0.biology.ucla.edu/structureHarvester/). The optimum value for *K* was defined based on the mean log-likelihood [L(K)] and the delta *K* (ΔK) statistic, according to the method proposed by Evanno et al. [26].

Population clusters, based on the STRUCTURE analysis; were compared through an analysis of molecular variance (AMOVA) [27]. This analysis was carried out using GenAIEx version 6 [28]. Based on the F statistics of the AMOVA, genetic differentiation among populations was calculated as Wright’s *F*_*ST*_ [29]. Gene flow (Nm) between population clusters was calculated as Nm = [0.25 (1- *F*_*ST*_)/(*F*_*ST*_)] [30]. Moreover, genetic data were subjected to a Genetic Barrier analysis to identify ecological barriers to gene flow among populations. This was performed based on Monmonier’s maximum difference algorithm, which treated geographical coordinates and genetic distances (Dice Coefficient) of each population as inputs. The Genetic Barrier analysis was carried out with the software BARRIER, ver. 2.2 [31].

The diversity parameters percentage of polymorphic loci, average number of alleles per locus, average effective number of alleles per locus, Shannon information index (*I*), and Nei’s genetic diversity (*He*) were calculated with GenAIEx version 6 [28]. These indexes were obtained for each of the genetic clusters formed by the STRUCTURE analysis. The Wilcoxon test with Bonferroni´s correction was performed to compare the diversity indexes between clusters (α = 0.05).

### Environmental niche modeling

To quantify the ecological differentiation, the environmental niche of the genetic clusters, derived from the STRUCTURE analysis, was modeled with Maxent 3.3.3 [16]. The coordinates of populations integrated into the genetic clusters were utilized to model the environmental niches. Bioclimatic variables 1–19 from Worldclim 1.4 [32] were used as predictors. Prior to any modeling step, a pairwise correlation matrix was calculated to identify possible collinearities existing among predictors. As a result, nine variables were discarded from the models because they were highly correlated with or more of the rest of the variables (Pearson’s correlation coefficient >0.85), leaving 10 bioclimatic variables. Variables included in the models were mean diurnal range (Bio2), temperature seasonality (Bio4), max temperature of the warmest month (Bio5), mean temperature of the wettest quarter (Bio8), mean temperature of the driest quarter (Bio9), mean temperature of the coldest quarter (Bio11), precipitation seasonality (Bio15), precipitation of the wettest quarter (Bio16), precipitation of the driest quarter (Bio17) and precipitation of the warmest quarter (Bio18). These environmental variables were downloaded from the website https://www.worldclim.org. Environmental niche modeling was carried out to predict the past, present, and future suitable areas for the distribution of the genetic clusters revealed by the STRUCTURE analysis.

The MaxEnt program offers five features: Linear (L), quadratic (Q), product (P), threshold (T) and hinge (H). These features were used to develop 25 MaxEnt models, based on different feature combinations (LQ, LQH, LQHP, and LQHPT) and regularization multipliers (1, 2, 5, 10, 15, and 20). This was performed by using the ENMeval package [33] in R software. Models were selected using two criteria: first, they were separated based on partial ROC tests [34], discarding non-significant models (P>0.1). Second, the models were sorted by the Akaike Information Criterion corrected for small sample sizes (AICc), choosing those with the lowest values as the final models [35].

Paleoclimatic data for the Last Interglacial (LIG; 120,000–140,000 years ago) and the Last Glacial Maximum (LGM; 22,000 years ago) were used to predict the past distribution. The LIG was utilized at a 2.5 arc-minutes of spatial resolution [36]. Meanwhile, the LGM was obtained based on the Community Climate System Model 4 (CCSM4), at a 30 arc-seconds resolution [37]. Simulations to estimate the present distribution were performed with the historical climate data for the period 1970–2000, at 30 arc-seconds resolution. Future climate data were obtained from projections of the MIROC-ES2L climate model [38], at 2.5 arc-minutes spatial resolution. Near and mid-century projections (2021–2040 and 2041–2060) were used, based on the Representative Concentration Pathway (RCP) 4.5. RCP 4.5 is the second-to-lowest emission scenario and could be considered to be a moderate mitigation scenario [39]. The models were constructed by using 50 replicate runs. Runs were evaluated by dividing the sampling points into 75/25% train/test, then estimated using the Area Under the Receiver Operating Curve (AUC). MaxEnt results were transformed into geographic maps showing the probability of occurrence from 0 to 1, where 1 indicates the maximum probability of occurrence and 0 indicates inadequate environmental conditions. All models were post-processed and visualized in ArcMap ver. 10.3 (ESRI, CA). The obtained environmental niches represent the modeled statistical association between the distribution of the genetic clusters and the climatic conditions.

**Fig 2:**
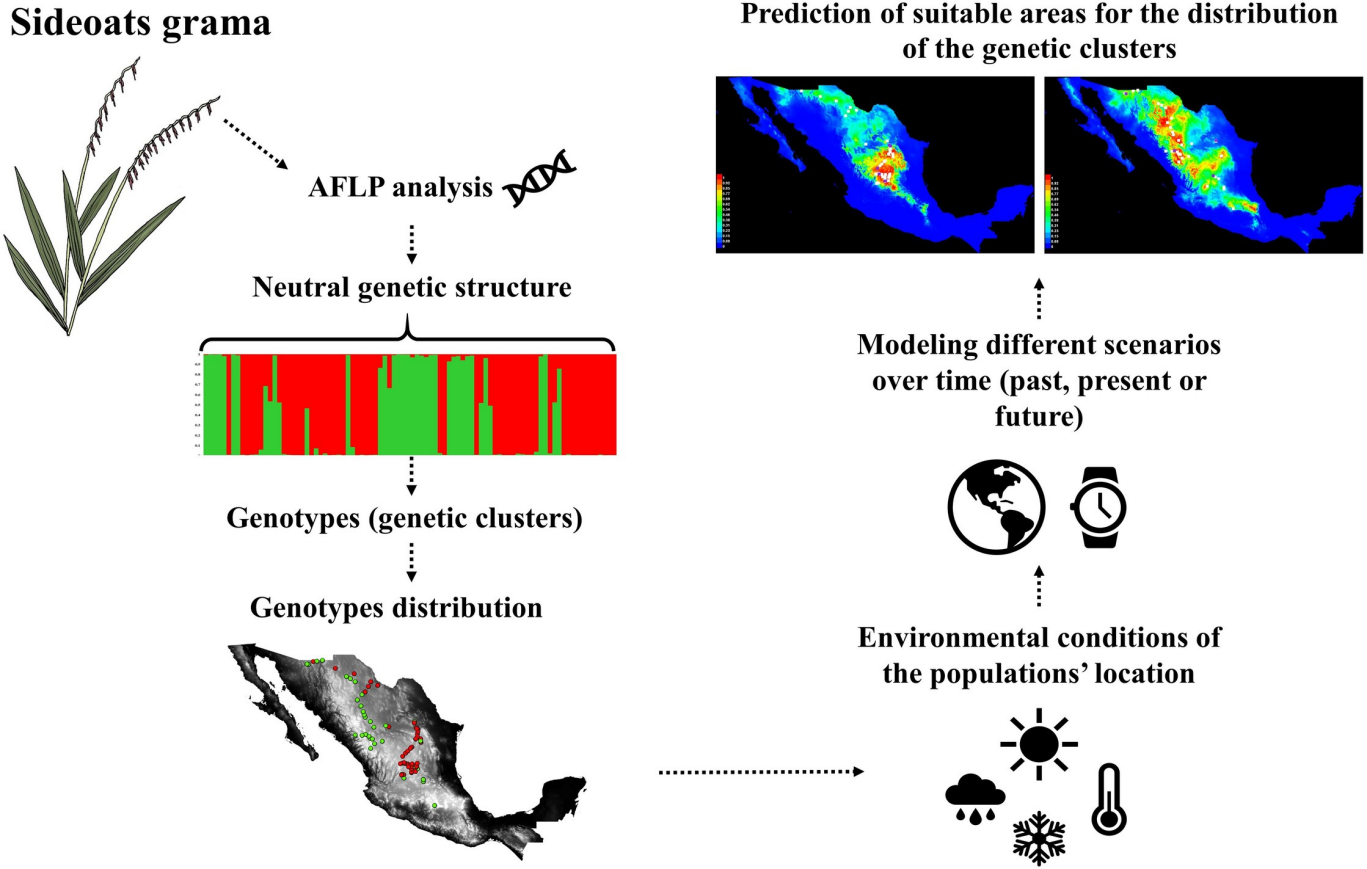
Summary of this study´s methodological approach. We used the information of AFLP molecular markers to quantify the number of genetic clusters existing within sideoats grama (*Bouteloua curtipendula*) populations in Mexico. Subsequently, we combined genotype and environmental data to simulate different scenarios over time (past, present and future) to determine the paleoclimatic interactions that have influenced the genetic structure within the species. This information was also used to predict suitable areas where each genotype can be used for restoration, in the present and future climate change scenarios.

The niche identity test was carried out to compare the environmental niches of the two genetic clusters. This test assessed if the suitable areas for the distribution of the two clusters overlap, in the past, present and future scenarios. The niche identity test was conducted through the ENM Tools software package in R, ver. 4.0.4. To determine significance for the niche identity test, we ran 100 permutations in which sample labels were randomized to generate a null distribution of the identity statistics, Schoener’s D [40] and Warren’s I [41]. If the empirical value of these statistics fell below the 95% confidence interval of the null distribution, we considered the niches of the two clusters to be significantly differentiated. Niche overlap measured as Schoener’s D (*SD*) and Warren’s I (*WI*) ranges from zero (no overlap) to one (total overlap). The methodological approach used in this study is summarized in Fig 2.

## Results

### Genetic structure

**Fig 3:**
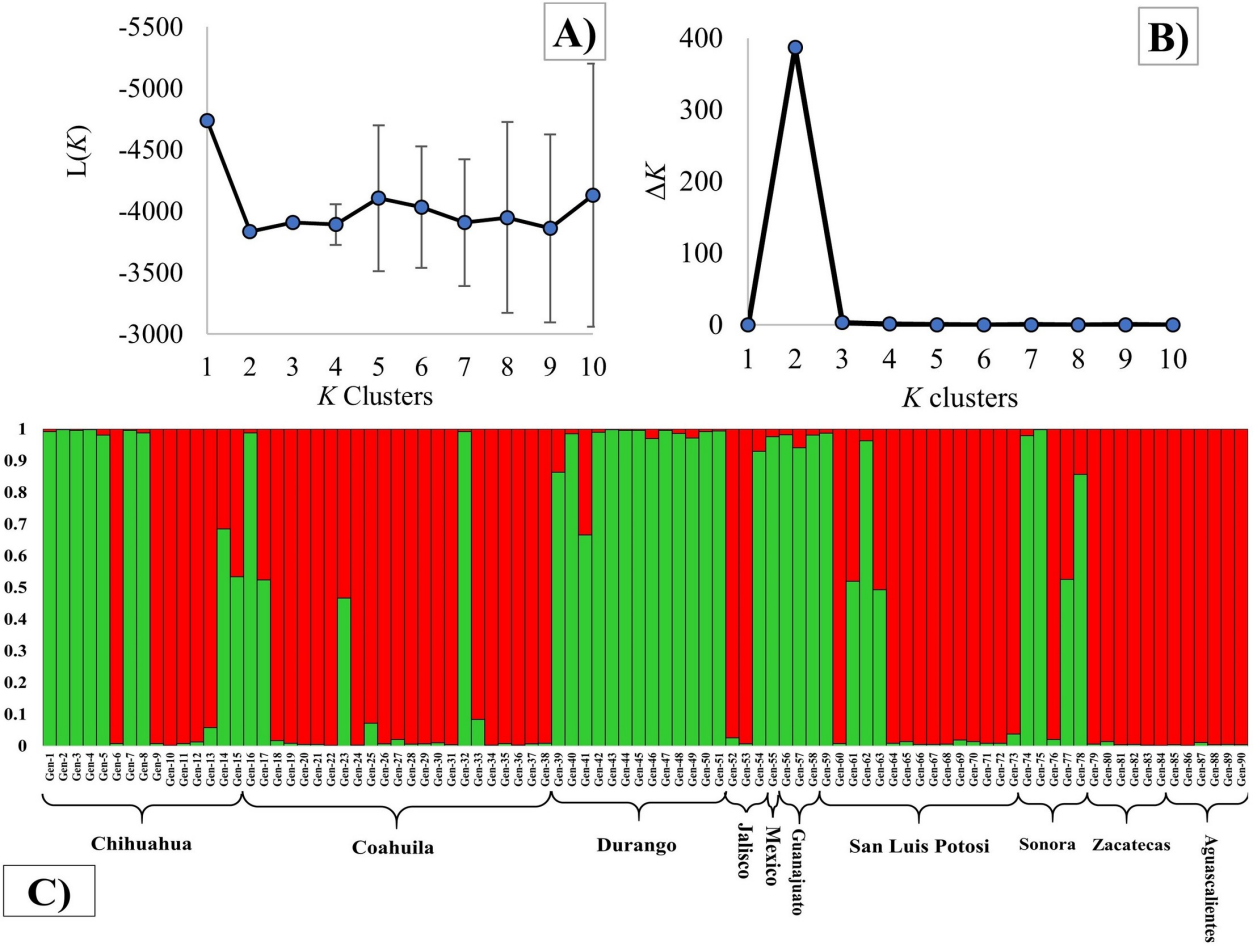
A) Mean log-likelihood [*L*(*K*)] and B) Evanno’s delta *K* (ΔK) values for AFLP based on STRUCTURE analysis of 85 sideoats grama (*Bouteloua curtipendula*) populations from 10 states of Mexico. *K* values tested were from 1 to 10. C) Barplots represent the estimated membership probability (y axis) of a population that belongs to a specific cluster (indicated by specific color).

The AFLP analysis generated 281 fragments, with 36.5% of them being polymorphic. The STRUCTURE analysis divided the sideoats grama populations into two genetic clusters, since *K* = 2 obtained the highest L(*K*) and Δ*K* values (Fig 3).

The AMOVA revealed differences between the clusters (p = 0.0003). Although the partitioning of the total AFLP variation showed that only 6.3% of the variation was explained by the differences between the clusters and, most of the genetic variation was presented within clusters (93.7%). Accordingly, the gene flow (Nm) between genetic clusters was 3.73 and the *Fst* 0.18, indicating a relatively high genetic exchange and low differentiation between clusters. In addition, the population clusters, grouped by the STRUCTURE analysis, showed no differences (p<0.05) in all of the diversity parameters evaluated (Table 1).

**Table 1:** Descriptive statistics of genetic diversity within two population clusters of sideoats grama (*Bouteloua curtipendula*).

Genetic cluster	Percentage of polymorphic loci	Average number of alleles per locus	Average effective number of alleles per locus	*I*	*H*_*e*_
Cluster I	37.0	1.34	1.20	0.181	0.120
Cluster II	36.7	1.33	1.18	0.176	0.115

*I* = Shannon Information index; *H*_*e*_ = Nei’s genetic diversity.

**Fig 4:**
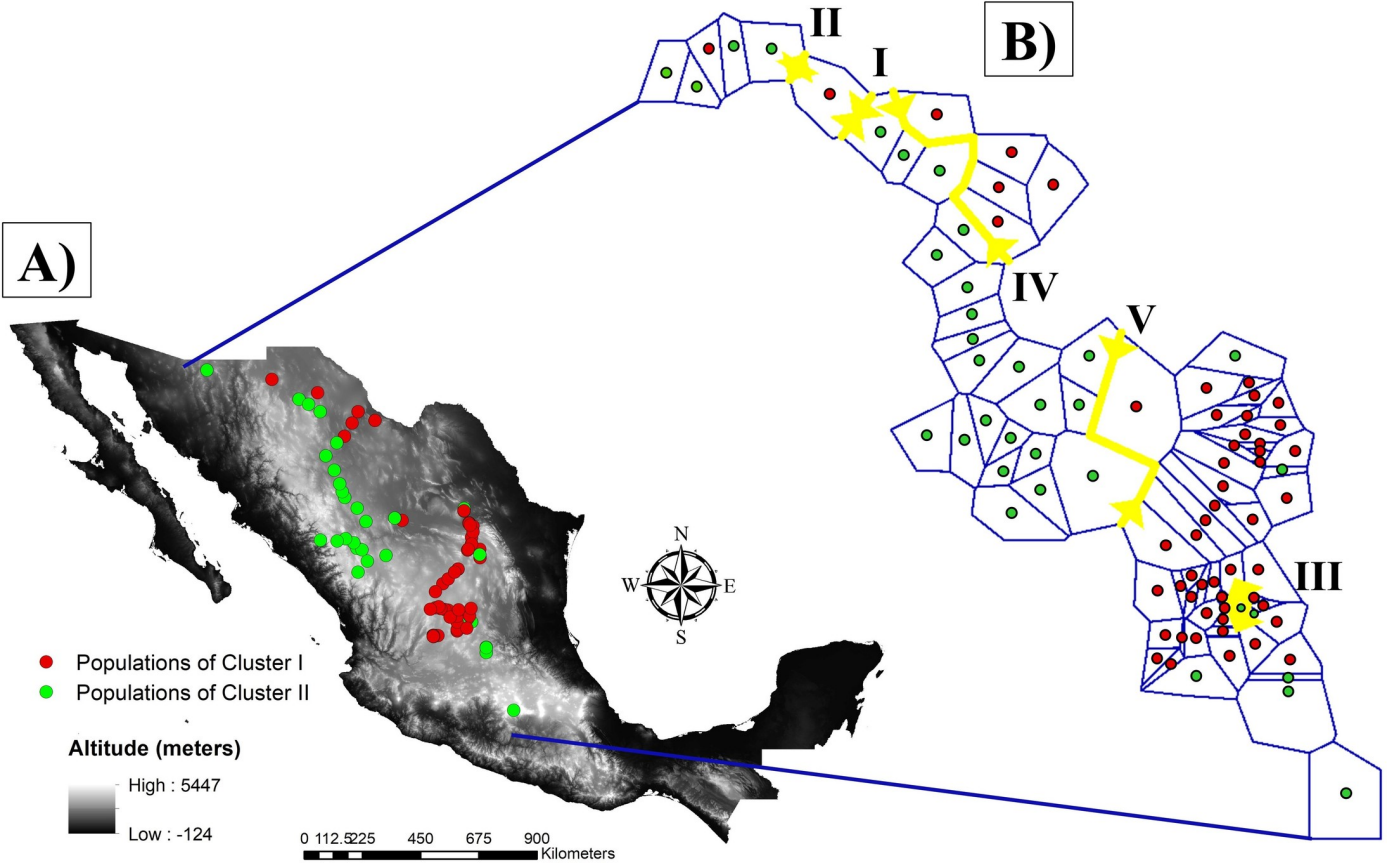
Genetic barriers predicted by BARRIER analysis among 85 sideoats grama (*Bouteloua curtipendula*) populations, divided into two clusters by STRUCTURE analysis. A) STRUCTURE clustering pattern in a geographical context. B) Genetic barriers (red lines) and Voronoi’s hypothetical population boundaries (blue lines). The analyses were performed base on 281 AFLP fragments. Digital Elevation Model from INEGI (https://www.inegi.org.mx).

Despite the high gene flow and the low differentiation found between clusters, as revealed for Nm and *Fst*, BARRIER analysis identified five genetic barriers to gene flow (Fig 4). The genetic barriers mainly isolated the populations located in the semi-arid region close to the Sierra Madre Occidental to the rest of the populations. Results from BARRIER analysis agree with the clustering patterns obtained in STRUCTURE analysis since both analyses generated similar clustering patterns.

### Environmental niche of the genetic clusters

Environmental niche modeling was conducted by using 53 sampling localities for Cluster I and 37 for Cluster II, according to the clustering pattern revealed by the STRUCTURE analysis. The past, present and future environmental niche models (ENMs) of clusters I and II were supported by AUCs higher than 0.92 and 0.82, respectively. The contribution of the variables varied among environmental niche models. However, there were variables of great contribution for the past, present and future ENMs of both clusters. Mean temperature of the driest quarter, mean temperature of the coldest quarter and precipitation of the wettest quarter were variables of the highest percentage contribution for the past, present and future ENMs of Cluster I. Mean temperature of the wettest quarter and mean temperature of the coldest quarter were predictors of the highest percentage contribution on the ENMs of Cluster II (Table 2).

**Table 2:** Contribution (%) of 10 bioclimatic variables to the environmental niche models for two genetic clusters of sideoats grama (*Bouteloua curtipendula*), at five different intervals of time.

ID	Variable	LIG		LGM		Present (1970–2000)		Near future (2021–2040)		Mid-century (2041–2060)	
		Cluster I	Cluster II	Cluster I	Cluster II	Cluster I	Cluster II	Cluster I	Cluster II	Cluster I	Cluster II
Bio2	Mean diurnal range	9.9	0.3	14.4	3.6	1.7	23.7	3.2	0.4	3.5	23.9
Bio4	Temperature seasonality	12.2	6.9	2.7	2.4	15.7	0.5	12.3	2.2	10.3	0.5
Bio5	Max temperature of warmest month	0.1	0.5	0.0	0.1	3.0	1.2	9.5	10.7	8.2	1.5
Bio8	Mean temperature of wettest quarter	0.8	7.0	7.4	62.4	11.5	20.5	3.3	7.1	7.8	10.8
Bio9	Mean temperature of driest quarter	14.7	3.0	26.3	7.6	28.6	6.2	29.2	48.8	21.3	4.3
Bio11	Mean temperature of coldest quarter	29.0	60.6	3.4	11.9	10.8	42.9	15.8	0.7	18.6	53.1
Bio15	Precipitation seasonality	3.6	8.0	11.6	3.8	0.2	1.8	2.7	0.3	0.4	1.5
Bio16	Precipitation of wettest quarter	4.4	5.3	24.9	2.7	19.2	1.2	16.6	0.1	21.8	1
Bio17	Precipitation of driest quarter	17.3	0.8	9.1	4.4	3.9	0.0	2	2.6	4.2	0
Bio18	Precipitation of warmest quarter	8.1	7.6	0.2	1.1	5.5	2.0	5.4	27.0	4	3.3

LIG = Last inter-glacial (120,000–140,000 years ago). LGM = Last glacial maximum (21,000 years ago).

**Fig 5:**
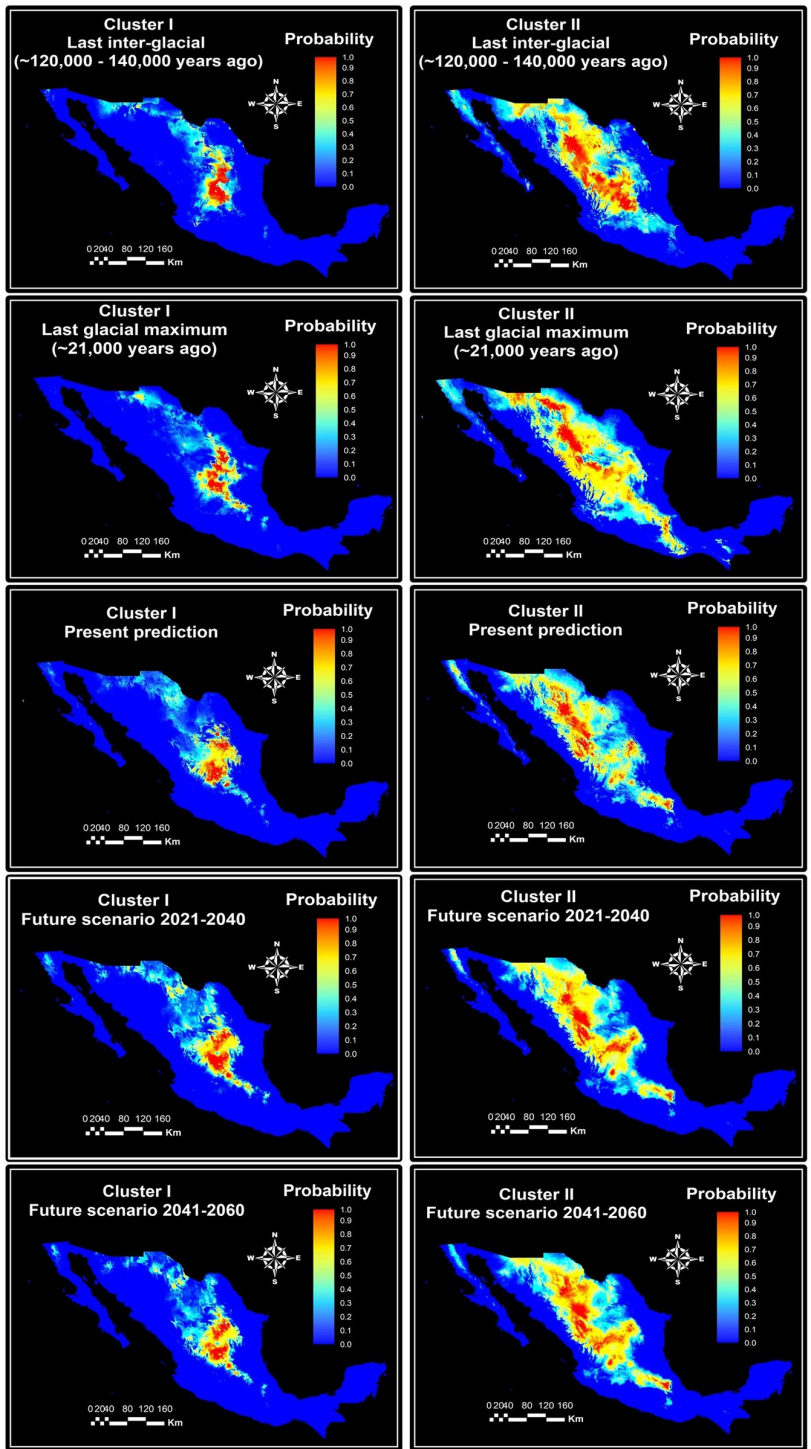
Past, present and future MaxEnt niche models for two genetic cluster of sideoats grama (*Bouteloua curtipendula*). Maps represent the average projections of the models to the last interglacial (120,000–140,000 years ago), last Glacial Maximum (22,000 years ago), historical data (1970–2000), near future (2021–2040) and mid-century (2041–2060). Genetic clusters were identified through AFLP markers and STRUCTURE analyses of 85 populations. Scale corresponds to probability of occurrence (habitat suitability).

The environmental niche of the two clusters resulted in a quite different geographical pattern, for the past, present and future scenarios (Fig 5). The present surface area with a high probability of occurrence (>0.8) was 39,372.2 and 72,425.6 Km^2^ for clusters I and II, respectively (Table 3). The niche identity tests revealed significant (p<0.05) differences between the present ENMs of the two clusters, according to the statistic for niche overlapping (*SD* = 0.48 and *WI* = 0.65). However, these results varied from the ones obtained in past and future ENMs. The surface area with a probability >80% for the distribution of Cluster I was similar for the LIG and present times, while Cluster II had approximately 18 thousand Km^2^ more surface area.

**Table 3:** Suitable areas and niche identity statistics for two genetic clusters of sideoats grama (*Bouteloua curtipendula*), based on MaxEnt environmental niche models for past, present and future scenarios.

Environmental niche model	Genetic cluster		Statistic for niche overlap	
	Cluster I (km^-2^)	Cluster II (km^-2^)	*SD*	*WI*
Last inter-glacial (120,000–140,000 years ago)	40,327.7	90,827.1	0.66*	0.80*
Last glacial maximum (21,000 years ago)	38,732.7	72,934.0	0.59*	0.76*
Present prediction (1970–2000)	39,372.2	72,425.6	0.48*	0.65*
Near future (2021–2040)	45,520.8	69,548.6	0.55*	0.74*
Mid-century (2041–2060)	40,225.7	76,753.5	0.57*	0.75*

Surface areas for a probability > 0.8. *SD* = Schoener’s [37] and *WI* = Warren’s [38] statistic for niche overlap between clusters. Niche overlap measured as Schoener’s D and Warren’s I ranges from zero (no overlap) to one (niche models identical).

*Indicates significant differences (p<0.05) between the environmental niches of the two clusters.

During the LGM, the surface area with a probability of occurrence >80% of Clusters I and II showed a reduction of 1,595 and 17,893 km^2^, compared with the LIG models. However, the surface area with a probability of occurrence >80% of both clusters were similar during the LGM and the present time. Likewise, environmental niches of clusters I and II were less distinct during the LGM, but similarly distinct to the present (Table 3).

Comparing present and future ENMs, the areas with a probability >80% for the distribution of Cluster I will increase 6,148 km^2^ during the near future (2021–2040) but will be similar to the present at the mid-century (2041–2060). In contrast, the surface areas with a probability >80% for the distribution of Cluster II will be 2,877 km^2^ less during the near future but 4,327 km^2^ greater at the mid-century.

**Fig 6:**
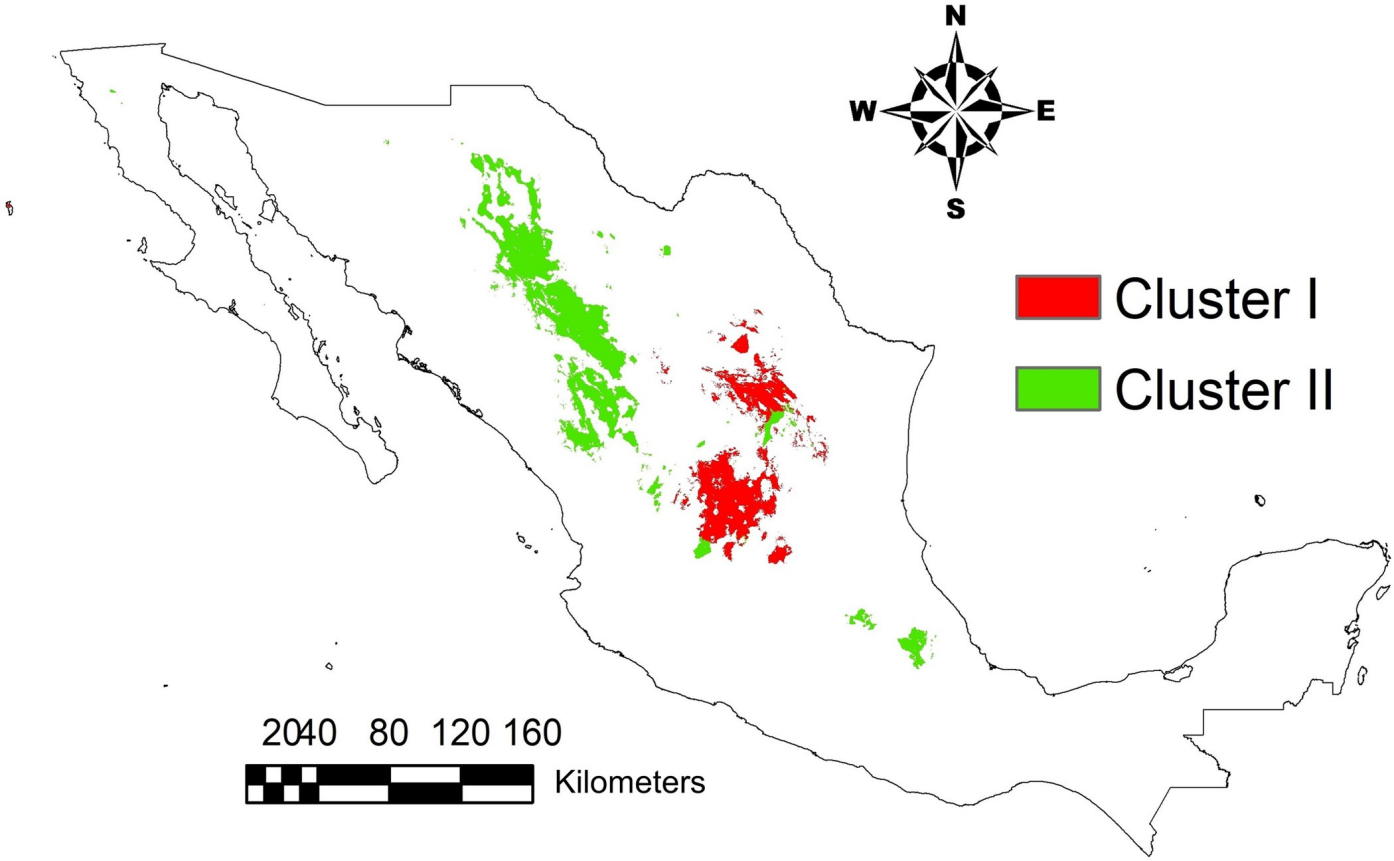
Areas with a probability of occurrence greater than 80% for the distribution two genetic clusters of sideoats grama (*Bouteloua curtipendula*), based on historical (1970–2000) bioclimatic variables as derived from environmental niche models in MaxEnt.

The present ENMs for the two clusters resulted in a quite distinct geographical pattern. The surface with a probability >80% for the distribution of Cluster I is mainly located in the central part of the country. For Cluster II, the surface with a probability >80% is mainly distributed in the north of the country, in the semi-arid region in proximity to the Sierra Madre Occidental (Fig 6).

**Fig 7:**
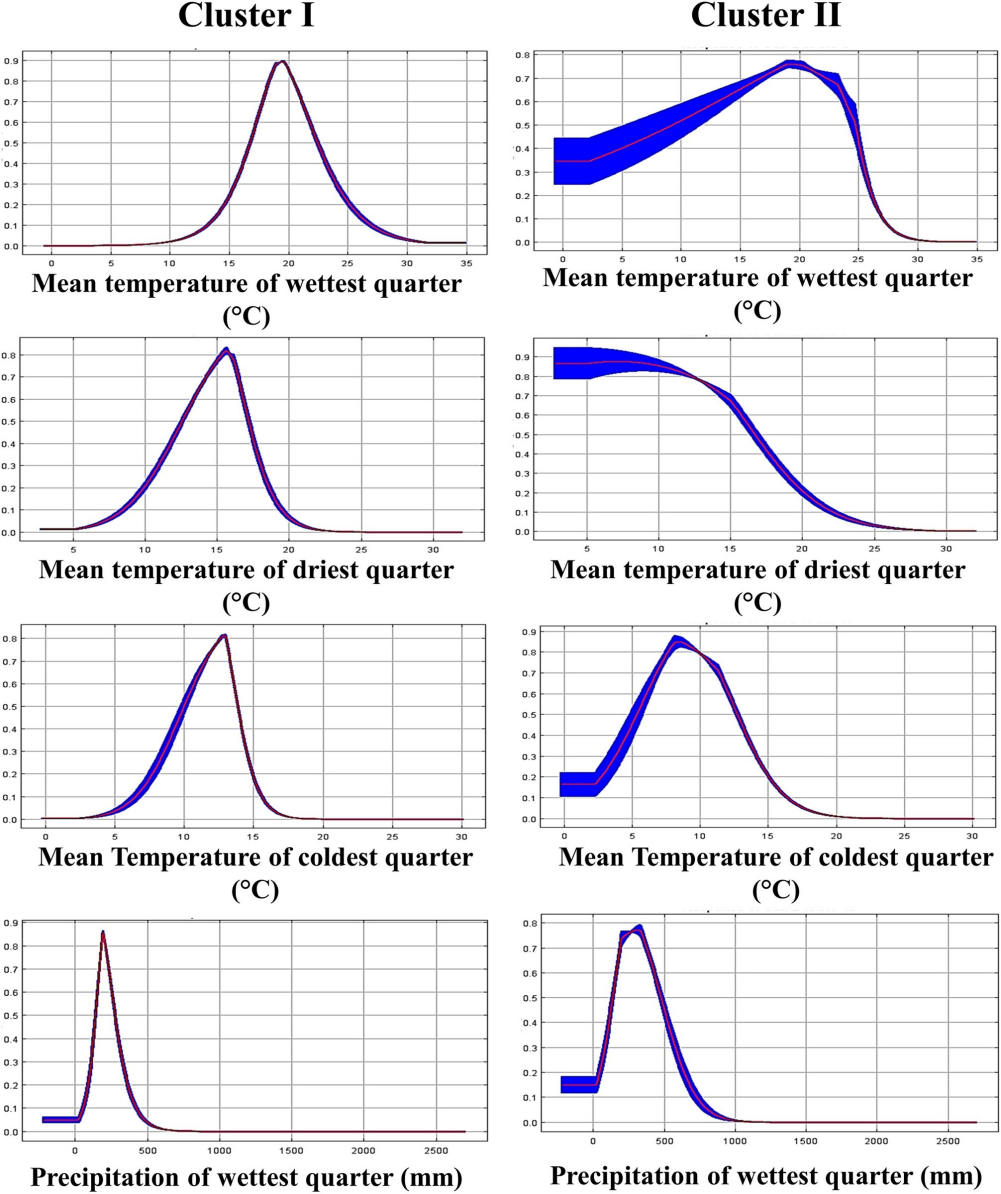
Response curves of the greatest contribution variables for the enviromental niche models of two genetic cluster of sideoats grama (*Bouteloua curtipendula*).

For a more detailed analysis, the response curves of some of the variables with the greatest contribution to the present ENMs are shown in Fig 7. According to these curves, the maximum probability of occurrence for both genetic clusters is close to 19°C of the mean temperature of the wettest trimester. However, populations from Cluster II may be adapted to areas with lower temperatures than those from Cluster I. The probability of occurrence for Cluster I at 10°C of the mean temperature of the wettest quarter is almost 0, while for Cluster II is 0.5. Cluster I reaches the highest probability of occurrence (0.88) in areas with 200 mm precipitation of the wettest quarter; however, its probability of occurrence declines to 0.2 at 100 mm. In contrast, Cluster II reaches the highest probability of occurrence (0.78) in areas with 300 mm precipitation of the wettest quarter and remains on 0.4 at 100 mm.

## Discussion

Population genetic structure can reveal the interactions of various evolutionary processes, such as population isolation and gene flow. According to the AFLP information and the STRUCTURE analysis, the sideoats grama populations may be divided into two genetic clusters. The reason for the divergence between clusters might be the variation in precipitation. The populations belonging to Cluster II are located in areas with lower precipitation, compared with Cluster I. The location of the populations of Cluster II average 389 mm of annual precipitation and 209 mm of precipitation during the rainfall season. Meanwhile, the location of the populations of Cluster I average 478 mm of annual precipitation and 295 mm of precipitation during the rainfall season, according to the data obtained from Worldclim. These results are in agreement with previous studies, which found that aridity can produce a genetic divergence among lineages of plant species [42–44].

The partitioning of the AFLP variation showed that the genetic differences between clusters explain only a small (6%) proportion of the total variation. This suggests there is a wide genetic variation within clusters and the possibility of selecting populations individually, instead of the whole genetic cluster. In grasses, differences within genetic clusters often explain only a small proportion of the total variation, due to the high genetic exchange commonly occurring among their populations [45, 46]. Accordingly, the gene flow (Nm) between genetic clusters was 3.73. This value of Nm can be considered high, compared with those obtained in previous research studies on grasses. For example, Coppi et al. [47] found a restricted gene flow (Nm = 0.497) among *Phragmites australis* populations. Furthermore, Zhang et al. [43] reported an Nm of 0.95 among *Festuca ovina* populations. Likewise, the *Fst* between clusters was 0.18, suggesting relatively low differentiation between clusters. Mitchell et al. [45] obtained a *Fst* of 0.02 among 85 Australian populations of *Microlaena stipoides*. The above mentioned studies were performed by using AFLP markers.

The lack of differences between clusters in all of the diversity parameters evaluated implicates that both clusters possess a similar level of genetic diversity. Cluster I and II presented a Shannon Information index of 0.181 and 0.176, respectively. Todd et al. [48] evaluated 56 accessions of *Panicum virgatum* and obtained an *I* value of 0.317. Likewise, Wanjala et al. [49] analyzed 281 cultivars of *Pennisetum purpureum* and found *I* values from 0.12 to 0.34. In comparison with these findings, the *I* values obtained in this study indicate an intermediate level of genetic diversity within the clusters.

The ENMs of both cluster distributions could be modeled with acceptable accuracy, since their AUCs were higher than 0.8 [16, 50]. ENMs provide preliminary evidence of niche specialization among the sideoats grama populations in Mexico. According to the present ENMs, Cluster II had 33,053.4 Km^2^ more surface area with a high probability of occurrence (>0.8) compared with Cluster I, suggesting that populations from Cluster II are adapted to a broader range of environmental conditions than populations from Cluster I. However, these results varied from the ones obtained in past and future ENMs. The surface area with a probability >80% for the distribution of Cluster I was similar for the LIG and present times, while Cluster II had approximately 18 thousand Km^2^ more surface area. The LIG was characterized with higher sea-level, CO_2_ concentrations and average global surface temperature than the Holocene [51, 52]. During this period, the temperature was 1.7°C higher on land and 0.8°C in the oceans, than in the pre-industrial Holocene [36]. This was a consequence of the higher isolation and the lower global ice volume [53, 54]. In North America, the climate became drier towards the late LIG with the rain season concentrated in the summer, producing an expansion of the C4 grasslands distribution [55]. The aforementioned is consistent with the result obtained in this study, where the suitable areas for the distribution of both genetic clusters were higher than the present. Furthermore, results from the LIG models suggest that populations of Cluster I may be better adapted to drought and high temperatures than Cluster II, because this cluster showed better adaptability to the LIG warm and dry conditions. The overlapping between the environmental niche of the two clusters was higher during the LIG, compared to the models corresponding to present time. This niche overlapping may have reinforced gene exchange between genetic clusters and influenced the low genetic differentiation between them. This is consistent with earlier findings that relate genetic structure with paleoclimatic patterns [9, 13].

Clusters I and II presented less surface area with a probability of occurrence >80% during the LGM compared with the LIG models, but similar to the present. Likewise, environmental niches of clusters I and II were less distinct during the LGM, but similarly distinct to the present. The changes in the environmental niches of the sideoats grama genetic clusters may be caused by the cooling period that occurred during the LGM. The LGM was a glacial period, caused by a reduction of atmospheric CO_2_ and sea level [36, 56]. The global cooling during the LGM generated a strong cooling in the Northern hemisphere [57]. Cluster II was more affected by the climate change caused by LGM, than Cluster I. The areas with a probability of occurrence greater than 0.8 for Cluster I increases in northern Mexico and decreases in the central part of the country. This may be produced for the precipitation patterns that occurred during this period. According to Metcalfe et al. [58], during the LGM, northern Mexico was notoriously wetter than the present due to an increased winter rainfall, while in the central part of the country less precipitation than at present occurred. Changes in surface area with a probability >80% are consistent with the environmental changes, which occurred during LGM.

In the study area, the evaluated future scenarios predict an increase in the precipitation of the wettest quarter in the near future and, a decrease during the mid-century period, compared with the present. This is in agreement with Seager and Vecchi [59], who anticipated that anthropogenic drying will reach the amplitude of natural decadal variability by mid-century, in North America. Likewise, Dai [60] pointed out that most of the Americas will experience severe droughts in the mid-century. Thus, populations from Cluster II appear to be more resistant to anthropogenic drying, since the suitable areas for the distribution of this cluster will increase at the mid-century.

The present ENMs for the two clusters resulted in a quite distinct geographical pattern. These results suggest the genetic clusters are ecologically distinct; hence, they can be considered as two distinct ecotypes. Each ecotype (genetic cluster) can be also considered to have its own conservation unit. Thus, restoration programs with sideoats grama should be implemented by using local germplasm from each ecotype. The aforementioned is consistent with previous studies suggesting restoration programs must be carried out using local genotypes, to preserve the genetic diversity and to guarantee a high probability of success [61]. However, most of the sideoats grama seeds used for restoration in Mexico are imported from the United States. For this reason, several breeding programs have been performed to select local outstanding genotypes of sideoats grama in the past years [2–5, 62]. Nevertheless, such programs have been mainly focused on agronomic traits and little attention has been paid to the genetic structure and environmental adaptation of the selected genotypes. In contrast, results from this study provide information on the environmental adaptability of sideoats grama populations in Mexico, which may be useful for future breeding programs. The AFLP markers examined in this study have revealed genetic clusters associated with geographical and climatic patterns. The high genetic diversity found within these clusters suggests the possibility of selecting outstanding populations from each cluster. Meanwhile, the niche specialization revealed by the ENMs, highlights the potential of selecting genotypes based on environmental adaptability.

## Conclusions

The sideoats grama populations analyzed in this study can be separated into two genetically and ecologically distinct clusters. One is mainly located in the central part of Mexico and the other predominantly in the north, in the semi-arid region near the Sierra Madre Occidental. The distribution pattern of these lineages can be partially explained by the paleoclimatic events that occurred during the LIG and LGM periods, which may have influenced the genetic exchange or isolation among populations.

Each genetic cluster has a different environmental niche and they can be considered as two distinct ecotypes. Therefore, we recommend each cluster to have its own conservation unit with potentially unique management needs. Accordingly, restoration programs with sideoats grama should be performed by using local germplasms from the environmental niche of each cluster. Predictions of future distributions suggest climate change will affect the distribution of the environmental niches of the two clusters. This information should be considered for future restoration programs. Finally, the high genetic diversity found within each cluster represents an opportunity to select outstanding germplasm, which may be used in future restoration programs. However, the results from this study need to be validated with reciprocal transplant trials, performed in the two environmental niches of the genetic clusters.

## Supporting information

S1 Table Coordinates of the geographic location of the collected samples from the 85 sideoats grama (*Bouteloua curtipendula*) populations. (XLSX)
